# Supplementation with *Enterococcus lactis* (SF68) and its association with biochemical parameters and inflammatory biomarkers related to renal impairment in dogs with chronic kidney disease

**DOI:** 10.1080/01652176.2026.2665483

**Published:** 2026-04-29

**Authors:** Ignacio López, Carmen Pineda, Antonio Camargo, Carolina Arenas, Ana Torrano, Griet Glorieux, Escolástico Aguilera-Tejero

**Affiliations:** a Department Medicina y Cirugía Animal, Hospital Clínico Veterinario, University of Cordoba, Cordoba, Spain; b Maimonides Institute for Biomedical Research in Cordoba (IMIBIC), Cordoba, Spain; c Lipids and Atherosclerosis Unit, Department of Internal Medicine, Reina Sofia University Hospital, Cordoba, Spain; d Department Medical and Surgical Sciences, University of Cordoba, Cordoba, Spain; e CIBER Fisiopatologia Obesidad y Nutricion (CIBEROBN), Instituto de Salud Carlos III, Madrid, Spain; f Hospital Valencia Sur-Anicura, Valencia, Spain; g Department Internal Medicine and Pediatrics, Nephrology Section, Ghent University Hospital, Ghent, East Flanders, Belgium

**Keywords:** Blood pressure, dog, indoxyl sulfate, kidney disease, microbiome, probiotic, *Ruminococcus gnavus*, symmetric dimethylarginine

## Abstract

To investigate the influence of probiotic supplementation on parameters of renal function, dogs with chronic kidney disease (CKD) received a commercial probiotic formulation containing *Enterococcus lactis* SF68 (*n* = 8) or placebo (*n* = 8) for 60 days. Gut microbiome was investigated by comparing with healthy dogs (*n* = 10). Blood biochemistry, urinalysis, inflammatory and oxidative markers, uremic toxins and blood pressure were monitored. Higher presence of *Lachnospiraceae* family, *Blautia* bacterial species and *Ruminococcus gnavus* group was observed in dogs with CKD when compared with healthy dogs. Adding the probiotic to the diet decreased the abundance of *Ruminococcus gnavus.* Probiotic treatment resulted in a significant reduction in plasma concentrations of symmetric dimethylarginine (SDMA), from 1.50 ± 0.18 to 1.35 ± 0.16 µmol/l (*p* = 0.008), and indoxyl sulfate (IxS), from 19.1 ± 6.8 to 12.8 ± 4.8 µmol/l (*p* = 0.04). Cytokine inflammatory markers did not show significant changes. An increase in urine protein-to-creatine ratio, 1.5 ± 0.6 vs 1.2 ± 0.5, and in systolic blood pressure, 163 ± 11 vs 144 ± 6 mmHg (*p* = 0.033), was observed in the placebo group but not in dogs receiving probiotic. In conclusion, feeding *Enterococcus lactis* SF68 to dogs with CKD results in changes in intestinal microbiota that are associated with a decrease in plasma concentrations of IxS and SDMA and a reduction in proteinuria and systolic blood pressure.

## Introduction

Chronic kidney disease (CKD) is a common condition in dogs that results in a progressive loss of kidney function and associated cardiovascular, hematological and skeletal complications (Perini-Perera et al. 2021). Current management of canine CKD involves dietary adjustments aimed preferentially to decrease protein and phosphorus intake (Polzin et al. 1984; Hall et al. 2018) and to reduce oxidative stress (Yu et al. 2006) in combination with a variety of drugs. These treatment approaches, while effective, do not achieve complete control of the disease and therefore additional therapeutic strategies should be investigated.

In human medicine, there is growing evidence that the gut microbiota of CKD patients is altered and that gut dysbiosis favors the synthesis of toxins with proinflammatory action (Nii-Kono et al. 2007; Barreto et al. 2009; Gross et al. 2015; Nangaku et al. 2015; Tang et al. 2015; Stubbs et al. 2016) that contribute to the progression of kidney disease (Evenepoel et al. 2009; Vaziri et al. 2013; Ramezani and Raj 2014; Bartochowski et al. 2022). Changes in the gut microbiota that result in an increase in gut-derived uremic toxins including indoxyl sulfate (IxS), *p*-cresyl sulfate (pCS), *p*-glucuronide (pCG), indole acetic acid (IAA) and trimethylamine-Noxide (TMAO) have also been reported in cats and dogs with CKD (Cheng et al. 2015; Summers et al. 2019).

There is abundant literature supporting the role of probiotic supplementation as an adjuvant therapy to improve the balance of the gut microbiota, contributing to intestinal barrier integrity and metabolic control in humans with kidney disease (Shariaty et al. 2017; Lim et al. 2021; Liu et al. 2024). In dogs, however, this subject has not been studied in depth. To our knowledge, there is only one published report in which probiotic supplementation with *Enterococcus lactis* was found to reduce the deterioration over time of glomerular filtration rate in dogs with CKD (Lippi et al. 2017).

In addition, several studies have reported physiological actions of Enterococcus *lactis* in dogs, including enhancement of mucosal and systemic immunity in puppies (Benyacoub et al. 2003) and limited but potentially helpful gastrointestinal effects in dogs with diarrhea (Simpson et al. 2009; Bybee et al. 2011; Torres-Henderson et al. 2017).

The aim of this study was to investigate the effects of a commercial probiotic formulation containing *Enterococcus lactis* (SF68) for companion animals on the gut microbiota profile of dogs with CKD and the influence of probiotic supplementation on parameters related to the severity of the disease.

## Materials and methods

### Animals

The study was conducted in dogs diagnosed with CKD IRIS (International Renal Interest Society) stage II and III, whose owners agreed to participate under informed consent. Dogs were recruited from the Hospital Clínico Veterinario of the University of Córdoba (Spain) and Hospital Veterinario Anicura Valencia Sur (Spain). Clients were offered diagnostic protocols, treatments and follow-ups free of charge. Ethical approval was granted through evaluation of the study by the Research Ethics Committee of the Hospital Clínico Veterinario of the University of Córdoba (Spain) (Registration Number 002, 25/03/2020).

Chronic kidney disease was diagnosed based on the dog's recent clinical and analytical history. All animals were clinically stable without acute signs of uremic crisis. Thus, only dogs with kidney disease in an IRIS stages II and III (with serum creatinine greater than 176,8 µmol/l and lower than 442 µmol/l; or symmetric dimethylarginine (SDMA) greater than 0.89 µmol/l and lower than 2.67 µmol/l) were included in the study. All dogs were free of other conditions that may have contributed to rapid evolution of kidney disease such as uncompensated heart disease, neoplasia, diabetes mellitus or advanced nephrotic syndrome. Dogs that were receiving medication for treatment of renal disease (angiotensin-converting enzyme inhibitors and angiotensin II receptor blockers) were maintained on the same medication throughout the study.

In addition to the study dogs, ten clinically healthy dogs fed a standard commercial diet donated feces that were used as a control for the study of gut microbiota. These samples were obtained with the informed consent of the dog owners.

### Study design

At the time of inclusion in the study all dogs were on a renal diet that had been fed for at least two months and they continued on the same diet throughout the study. When entering the study each dog was allocated to one of two groups:

Group 1 (Placebo, *n* = 8) a placebo was added on top of the food once a day for 60 days.

Group 2 (Probiotic, *n* = 8) the commercial probiotic Purina® Pro Plan® Veterinary Diets, Fortiflora® (Nestle Purina PetCare, St Louis, MO) was added on top of the food once a day for 60 days. Sixty days was considered sufficient to effect an influence on gut microbiome and to monitor evolution of biochemical parameters associated to renal disease.

As per label, placebo and probiotic had the same analytical composition (Supp Table 1) with the only difference that the placebo did not contain *Enterococcus lactis* SF68. To guarantee blinding, both supplements were prepared in capsules containing 0.5 g of powder that looked identical for placebo and probiotic. The content of the capsule was poured and mixed with the food and the owner was instructed to verify full consumption of the supplement. The owners and the veterinarians performing clinical examinations of the dogs were blinded separately to treatment.

Dogs were alternately assigned to treatment groups in the order of recruitment. Thus, the first dog was allocated by an independent person in Group 1, the second dog in Group 2, the third dog in Group 1, and so on. During the study dogs remained with the same diet they were receiving before recruitment because it was thought that changing the diet would introduce an element of variation in gut microbiome and also for logistic reasons related to reluctance of the owners to change diet. The diets were matched between groups: *Purina® Pro Plan® Veterinary Diets Canine NF Renal Function* (5 dogs Group 1, 4 dogs Group 2), *Hill's Prescription Diet k/d* (1 dog in each Group), and *Royal Canin® Renal* (2 dogs Group 1, 3 dogs Group 2). Composition of the renal diets is shown in Supp Table 2. As reported in the literature (Or et al. 2024), renal diets are quite homogeneous in their macro- and micronutrient formulation.

Physical exam, analytical control (complete blood count, blood biochemistry, inflammatory and oxidative profiles, and urine tests) and blood pressure measurement were performed in all dogs at the beginning of the treatment (day 0), one month later (day 30) and at the end of the study (day 60). Plasma uremic toxins were evaluated at the beginning (day 0) and at the end (day 60) of treatment. At these same time points (days 0 and 60) a study of the intestinal microbiota was performed. Body weight and body condition score (BCS) were measured weekly. Feed and water intake, appetite and characteristics of feces were monitored daily by the dog owner.

### Blood biochemical variables

Blood samples were obtained after an overnight fast. A complete blood count was obtained using an automated hematology analyzer (Sysmex XN-2000V; Sysmex Europe, SE, Norderstedt, Germany). A biochemical profile including plasma concentrations of urea, creatinine, SDMA, alanine aminotransferase (ALT), aspartate aminotransferase (AST), alkaline phosphatase (ALP), cholesterol, bilirubin, total proteins, albumin, globulins, calcium and phosphorus was measured (Beckman AU5811; Beckman Coulter Inc., Brea, California, USA). Plasma electrolytes (Na, K and Cl) were quantified with selective electrodes (Beckman AU5811; Beckman Coulter Inc., Brea, California, USA).

An inflammatory profile including plasma C-reactive protein (CRP), interleukins 2, 6, 8 and 10 (IL-2, IL-6, IL-8 and IL-10), tumor necrosis factor alpha (TNFα) and monocyte chemoattractant protein-1 (MCP-1) was obtained. The concentration of CRP was measured using a commercial ELISA kit (#ab157698, Abcam, Cambridge, UK). Quantification of all other cytokines was performed using a Milliplex^MAP^ Canine Cytokine Magnetic Bead Panel kit (EMD Millipore, Burlington, MA, USA).

The total antioxidant capacity (TAC) of plasma was measured using a TAC colorimetric assay kit based on the reduction of Cu^2+^ to Cu^+^ (OxiSelect, Cell Biolabs, Inc. San Diego, CA, USA) with the results being expressed as mM copper reducing equivalents.

For the quantification of uremic toxins, plasma was sent on dry ice to the Nephrology Laboratory of the Ghent University Hospital in Belgium for batch analysis. Total and free concentrations IxS, pCS, IAA, *p*-glucuronide (pCG), hippuric acid (HA) and uric acid (UA) were determined by liquid chromatography and fluorescence detection as previously described (Glorieux et al. 2021). In brief, for quantification of the total toxin concentrations, plasma samples were deproteinized by heat, centrifuged and filtered through an Amicon® Ultra 0.5 L (Merck Millipore Ltd. Carrigtwohill, Ireland) (molecular weight cut-off 30 kDa filter). For the free fraction, the untreated plasma was first filtered through the Amicon filter. The ultrafiltrate was transferred into an autosampler vial, and fluorescein was added as an internal standard. Analysis was performed by ultra-performance liquid chromatography with an Agilent 1290 Infinity device using an Agilent G1316C fluorescence detector (Agilent, Santa Clara, USA).

### Urinalysis

Five ml of urine were obtained by ultrasound-guided cystocentesis. Urinalysis included the following tests: urine test strips (Multistix® 10 SG, Siemens Healthcare Diagnostics Manufacturing Limited, Swords, Dublin, Ireland), measurement of urine specific gravity by refractrometry (Zuzi, Auxilab S.L., Navarra, Spain), quantitative determination of proteinuria with the urine protein to creatinine (UPC) ratio (BioSystems S.A., Barcelona, Spain), and a microscopic examination of urine sediment.

### Blood pressure measurement

Arterial blood pressure, systolic (SBP), diastolic (DBP) and mean (MBP), was measured by oscillometric method (PANI VET20, B. Braun VetCare, S.A., Melsungen, Germany). The cuff was placed around a forelimb. The size of the cuff (40% of limb perimeter) was standardized for each dog. The procedure involved obtaining five measurements with the dog quiet, relaxed and in lateral recumbency. The higher and lower values were discarded and the mean of the other three values was calculated.

### Evaluation of intestinal microbiota

Fecal samples were obtained by the owners on the same day that the dogs were subjected to evaluation. Fresh stool samples were collected in tubes containing a DNA stabilization buffer (Canvax Biotech, Spain). Upon arrival at the hospital, samples were frozen at −80 °C until analysis.

Samples were processed to determine the intestinal microbiota profile of the dogs by sequencing variation in the 16S microbial ribosomal RNA (rRNA) gene. DNA extraction from feces was performed using the QIAamp DNAStool Mini Kit Handbook (QIAGEN, Hilden, Germany) following the manufacturer's instructions. The intestinal microbiota was examined through 16S metagenomics on the Illumina MiSeq platform (Illumina, San Diego, CA, USA) and the data processed with Quiime2 software as previously described (Santos-Marcos et al. 2020). Further, sequencing data were analyzed and visualized using QIIME2 (Bolyen et al. 2019), using the DADA2 method (Callahan et al. 2016). Bacterial richness and diversity across the samples were calculated using the observed OTUs, Shannon, Simpson and Faith_pd indexes (Hammer et al. 2001). Principal component analysis of community structure (beta-diversity) was done using the Jaccard, Bray-Curtis and UniFrac (unweighted and weighted) distance metrics (Lozupone and Knight 2005) and analyzed by permutational multivariate ANOVA (PERMANOVA). Pre vs post treatment alpha- and beta-diversity was analyzed by qiime longitudinal pairwise-differences and qiime longitudinal pairwise-distances qiime2 plugins. Taxonomy was assigned to the high-quality reads using a q2‐feature‐classifier (Bokulich et al. 2018), with a sequence identity threshold of 99%, and by interrogating the sequences using the SILVA database (Quast et al. 2013), and relative abundance was calculated by dividing the number of reads of each bacterial taxon by the total number of reads. After filtering, the high-quality reads of the samples ranged from 44,594 to 78,796 sequence counts. Taxonomy was assigned to the high-quality reads using a q2‐feature‐classifier (Bokulich et al. 2018), with a sequence identity threshold of 99%, and by interrogating the sequences using the SILVA database (Quast et al. 2013), and relative abundance was calculated by dividing the number of reads of each bacterial taxon by the total number of reads. Microbiome Analyst 2.0 was used to compare groups at baseline and visualize the results, using a taxonomic heat tree and the Wilcox test as statistical analysis (Lu et al. 2023). In this analysis, a cut-off for exclusion was fixed to exclude bacterial taxa that were not present in most of the samples: only bacterial taxa containing sequence reads in at least 75% of the total samples were considered.

### Statistics

To estimate sample size, a power analysis was conducted with the G-Power Software (Buchner et al. Heinrich-Heine-Universität Düsseldorf 2025) using creatinine and SDMA as the reference variables. The following parameters were used: power = 0.8, significance level = 0.05, standard deviation (SD) = 35.4 µmol/l for creatinine and 0.25 µmol/l for SDMA. To detect differences among groups of 44.2 µmol/l for creatinine and 0.30 µmol/l for SDMA (that would correspond with 15%–20% changes in glomerular filtration rate), a minimum of seven to eight animals per group was estimated necessary. The SD values and the targeted differences in creatinine and SDMA were obtained from the literature (Kim et al. 2020).

Statistical analysis was performed using the software IBM Statistics SPSS 25 (Armonk, NY, USA). Epidemiological data of the dogs were grouped into contingency tables and analyzed using Fisher's exact test. Relationships between discrete variables were performed using a Chi-square test. Comparison of baseline data (day 0) between both groups was performed by unpaired *t*-tests. The response to treatment was assessed by comparing the values obtained at days 0, 30 and 60 in each animal using paired *t*-tests. Longitudinal changes in fecal microbiome induced by probiotics were analyzed by Wilcoxon range test. *p* values were not corrected for multiple testing. Results are presented as mean ± standard error. Significance level was set at *p* < 0.05.

## Results

### Animals

Epidemiological data of the dogs that participated in the study are shown in Table 1. Dogs assigned to the probiotic group were slightly older than dogs assigned to the placebo group, but the difference was not significant (*p* = 0.121). In addition, no significant differences in sex, primary disease leading to CKD or pharmacologic treatment were found when comparing both groups.

**Table 1.** Epidemiological data of dogs.

Parameters	PLACEBO (*n* = 8)	PROBIOTIC (*n* = 8)
**Age** (years) (mean ± SE)	5.7 ± 1.1	9.0 ± 1.7
**Sex**		
Male	2/8 (25%)	2/8 (25%)
Female	6/8 (75%)	6/8 (75%)
**Primary disease leading to CKD**		
Unknown	4/8 (50%)	4/8 (50%)
Leishmaniasis	2/8 (25%)	3/8 (38%)
Immune glomerulonephritis	2/8 (25%)	1/8 (13%)
**Treatment**		
ACEIs/ARB II	5/8 (63%)	6/8 (75%)

ACEIs = angiotensin-converting enzyme inhibitors, ARB II = angiotensin II receptor blockers, CKD = chronic kidney disease.

### Physical parameters

No changes in feed and water intake, and general well-being were observed during the study in any group. Subjective assessment identified an improvement in stool consistency in dogs treated with probiotics. The results of the physical exam did not vary at the different time points. No significant differences between groups were observed in body weight at day 0 (*p* = 0.324). Body weight did not change in any group after treatment: placebo, 21.9 ± 3.6 kg (day 0) vs 22.8 ± 3.9 kg (day 60) and probiotic: 16.3 ± 4.1 kg (day 0) vs 16.1 ± 4.0 kg (day 60). Correspondingly, body condition score was similar in both groups and remained unchanged during the study: placebo, 4.8 ± 0.4 (day 0) vs 5.0 ± 0.3 (day 60) and probiotic, 4.5 ± 0.4 vs 4.6 ± 0.3.

### Biochemical variables

**Figure 1.**
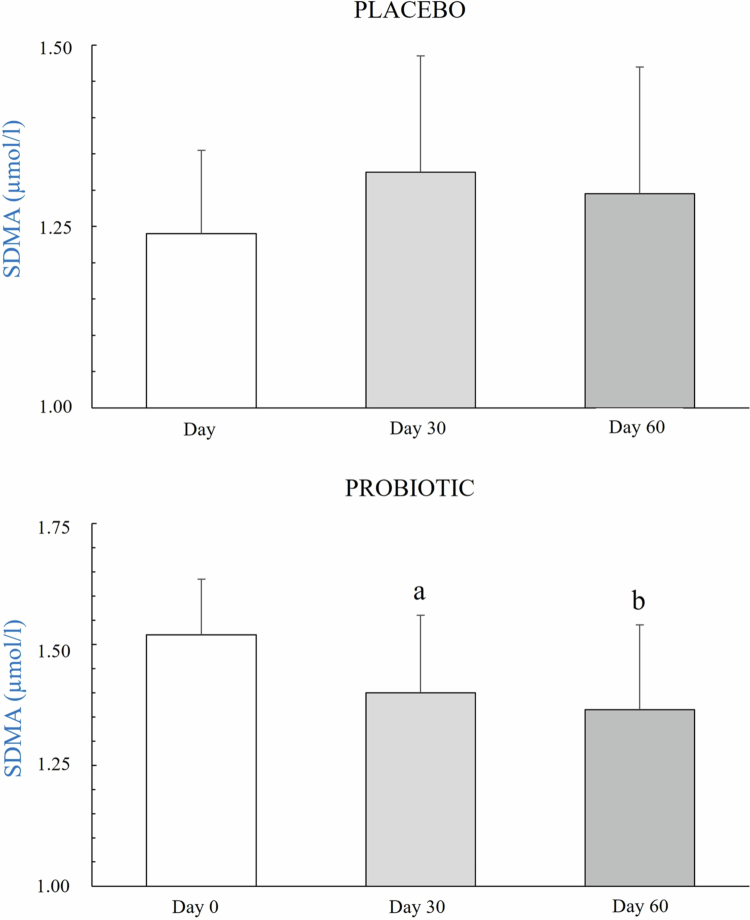
Plasma concentrations of SDMA along time (day 0, day 30 and day 60) in dogs with chronic renal disease treated with probiotic or placebo. ^a^*p* = 0.031 vs day 0 and ^b^*p* = 0.008 vs day 0.

Blood biochemistry profile at the beginning of the study was characteristic of dogs with CKD with increased values of urea, creatinine and SDMA. No significant differences were found between groups at day 0 in any of the parameters under study. No changes in urea, creatinine or SDMA were observed during the course of the study in dogs receiving the placebo. In contrast, probiotic administration resulted in a significant decrease in plasma concentration of SDMA both at day 30, 1.38 ± 0.19 µmol/l, and day 60, 1.35 ± 0.16 µmol/l, *p* = 0.031 and *p* = 0.008, respectively, when compared with day 0, 1.50 ± 0.18 µmol/l (Figure 1). Plasma cholesterol concentrations showed a non-significant trend to increase along the study in both groups and the differences reached significance in the probiotic group although the final values were lower in the probiotic than in the placebo group. No significant changes in any other biochemical, mineral or electrolyte parameter were detected in any of the groups (Table 2).

**Table 2.** Blood biochemistry values in the study groups (mean ± SE).

	PLACEBO (*n* = 8)			PROBIOTIC (*n* = 8)			Reference range
	Day 0	Day 30	Day 60	Day 0	Day 30	Day 60	
**Urea (mmol/l)**	14.1 ± 3.3	11.8 ± 2.2	12.6 ± 2.3	15.3 ± 2.4	18.0 ± 3.0	17.1 ± 3.6	3.5–9.8
**Creatinine (µmol/l)**	185.6 ± 17.7	185.6 ± 26.5	185.6 ± 26.5	176.8 ± 26.5	185.6 ± 26.5	185.6 ± 35.4	44.2–132.6
**Total proteins (g/l)**	59.3 ± 3.0	59.3 ± 2.8	58.8 ± 2.7	59.9 ± 2.3	58.5 ± 1.9	58.5 ± 2.4	48.0–78.0
**Albumin (g/l)**	24.5 ± 2.6	24.9 ± 2.2	24.5 ± 2.4	25.1 ± 1.7	24.5 ± 2.0	23.9 ± 2.3	27.0–41.0
**ALT (µkat/l)**	0.99 ± 0.36	0.75 ± 0.17	0.750 ± 0.18	1.00 ± 0.24	0.96 ± 0.32	0.64 ± 0.09	0.44–1.51
**AST (µkat/l)**	0.48 ± 0.05	0.57 ± 0.05	0.51 ± 0.06	0.62 ± 0.04	0.54 ± 0.04	0.58 ± 0.06	0.27–1.51
**ALP (nkat/l)**	3.13 ± 1.63	2.83 ± 1.36	3.39 ± 2.03	1.05 ± 0.44	1.26 ± 0.73	0.83 ± 0.27	0.22–1.79
**Cholesterol (mmol/l)**	7.78 ± 0.85	9.41 ± 1.19	9.03 ± 0.88	7.03 ± 0.93	8.09 ± 0.98*	8.17 ± 0.85*	2.90–8.43
**Tryglicerides (mmol/l)**	0.91 ± 0.17	0.98 ± 2.09	0.91 ± 0.23	0.98 ± 0.25	0.82 ± 0.16	0.98 ± 0.27	0.38–1.54
**tCa (mmol/l)**	2.50 ± 0.07	2.55 ± 0.07	2.52 ± 0.07	2.40 ± 0.09	2.57 ± 0.07	2.52 ± 0.07	2.05–2.97
**P (mmol/l)**	1.71 ± 0.16	1.84 ± 0.19	1.71 ± 0.19	1.87 ± 0.23	1.87 ± 0.23	1.84 ± 0.19	0.87–2.16
**Na (mmol/l)**	148.4 ± 1.2	148.3 ± 1.5	146.9 ± 0.6	149.4 ± 0.7	149.0 ± 0.7	147.4 ± 1.7	142–153
**K (mmol/l)**	5.0 ± 0.2	5.1 ± 0.2	5.0 ± 0.1	4.9 ± 0.3	4.9 ± 0.2	5.5 ± 0.3	3.9–5.6
**Cl (mmol/l)**	114.3 ± 1.7	114. 1 ± 1.8	113.4 ± 1.2	112.8 ± 2.0	110.9 ± 1.2	112.1 ± 1.1	105–121

*p < 0.05 vs day 0.

ALP = alkaline phosphatase, ALT = alanine aminotransferase, AST = aspartate aminotransferase, Cl = chloride, K = potassium, Na = sodium, P = phosphorus, tCa = total calcium.

### Inflammatory and oxidative profile

Values of IL2, IL10 and TNFα were below the detection limits of the assay at all time points which may reflect a technical limitation of the measurement. At day 0, plasma concentrations of MCP1 and IL6 showed a non-significant trend to be higher in the probiotic group, while IL8 values showed a non-significant trend to be higher in the placebo group. No statistically significant changes in the plasma levels of these cytokines were observed in either study group. In dogs receiving the probiotic, TAC showed a non-significant trend to increase at the end of the study, and the differences approached the significance level (*p* = 0.071). By contrast, TAC showed a non-significant trend to decrease in the placebo group (Table 3).

**Table 3.** Plasma cytokin levels in the study groups (mean ± SE).

	PLACEBO (*n* = 8)			PROBIOTIC (*n* = 8)		
	Day 0	Day 30	Day 60	Day 0	Day 30	Day 60
**MCP1 (pmol/l)**	38.2 ± 2.6	36.7 ± 5.2	87.6 ± 48.3	73.9 ± 25.6	52.9 ± 9.5	56.0 ± 9.7
**IL6 (pmol/l)**	1.80 ± 0.34	2.97 ± 1.81	3.43 ± 2.02	5.39 ± 2.74	2.39 ± 1.30	3.96 ± 1.30
**IL8 (pmol/l)**	1814 ± 770	1295 ± 437	1048 ± 285	1111 ± 218	1279 ± 260	1070 ± 223
**CRP (pmol/l)**	35.0 ± 26.0	22.6 ± 10.0	18.3 ± 9.0	18.3 ± 6.0	35.0 ± 23.0	22.6 ± 11.0
**TAC (mmol/l)**	21.3 ± 3.4	18.2 ± 1.2	18.9 ± 1.0	14.3 ± 0.7	16.3 ± 1.1	17.5 ± 1.7

CRP = C-reactive protein, IL6 = interleukin 6, IL8 = interleukin 8, MCP1 = monocyte chemoattractant protein-1, TAC = total antioxidant capacity.

### Uremic toxins

**Figure 2.**
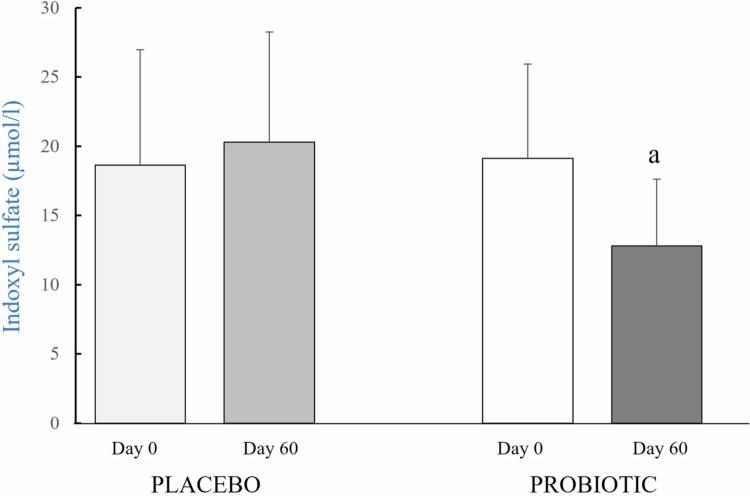
Plasma concentrations of indoxyl sulfate at the beginning (day 0) and at the end (day 60) of the study in dogs with chronic renal disease treated with probiotic or placebo. ^a^*p* = 0.04 vs day 0.

Total plasma concentrations of IxS and pCS showed a non-significant trend to increase in the placebo group reflecting the progression of CKD. However, both IxS and pCS decreased in the dogs receiving the probiotic, and the difference was significant (*p* = 0.04) in the case of IxS, 12.8 ± 4.8 µmol/l at day 60 vs 19.1 ± 6.8 µmol/l at day 0 (Figure 2). The levels of HA and UA did not change throughout the study in either group (Table 4). Values for IAA and pCG were below the detection limit of the assay.

**Table 4.** Total concentrations plasma *p*-cresyl sulfate (pCS), hippuric acid (HA) and uric acid (UA) in the study groups (mean ± SE).

	PLACEBO (*n* = 8)			PROBIOTIC (*n* = 8)		
	Day 0	Day 60		Day 0	Day 60	
**pCS (µmol/l)**	0.425 ± 0.053	0.478 ± 0.053		0.478 ± 0.053	0.425 ± 0.053	
**HA (µmol/l)**	14.51 ± 7.81	13.95 ± 7.81		15.63 ± 8.93	14.51 ± 8.93	
**UA (µmol/l)**	48.77 ± 4.76	49.37 ± 4.76		51.75 ± 4.16	52.34 ± 4.76	

### Urinary parameters

Urine specific gravity, which was similar at day 0 in both groups, 1016 ± 2 and 1016 ± 3, did not change during the study. UPC was above the reference range in both groups at the beginning of the study and showed a non-significant trend to increase in the placebo group, from 1.2 ± 0.5 (day 0) to 1.4 ± 0.5 (day 30) and 1.5 ± 0.6 (day 60). However, in the probiotic group, UPC remained stable, 1.0 ± 0.3, 1.1 ± 0.3 and 0.9 ± 0.3 at day 0, day 30 and day 60, respectively.

### Arterial blood pressure

Systolic blood pressure showed a non-significant trend to increase in the placebo group at 30 days and was significantly higher at 60 days versus day 0 (163 ± 11 mmHg vs 144 ± 6 mmHg, *p* = 0.033). In contrast, no increase in blood pressure was found in the probiotic group during the time of the study (Table 5).

**Table 5.** Systolic (SBP), diastolic (DBP) and mean (MBP) values of blood pressure in the study groups (mean ± SE).

	PLACEBO (*n* = 8)			PROBIOTIC (*n* = 8)		
	Day 0	Day 30	Day 60	Day 0	Day 30	Day 60
**SBP (mmHg)**	144 ± 6	148 ± 10	163 ± 11*	144 ± 9	141 ± 6	140 ± 6
**DBP (mmHg)**	96 ± 7	91 ± 9	100 ± 9	84 ± 3	91 ± 4	91 ± 2
**MBP (mmHg)**	106 ± 7	105 ± 8	112 ± 8	103 ± 5	105 ± 3	105 ± 2

*p < 0.05 vs day 0.

### Intestinal microbiota

**Figure 3.**
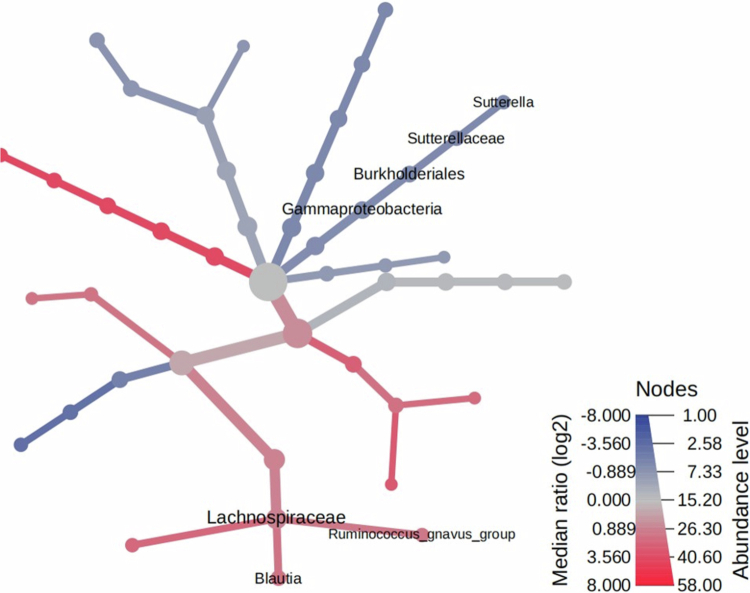
Taxonomic heat tree comparing the abundance of taxa in the intestinal microbiome in dogs with chronic renal disease, at the beginning of the study and a control group of healthy dogs.

In a first step, the microbiota of clinically healthy and CKD dogs was compared. While we found higher observed OTUs in dogs with CKD (*p* = 0.023), we did not observe any differences in Shannon or Simpson indexes. Moreover, we did not find differences in Fatih_pd index, considering this latter the bacterial phylogeny. Regarding beta-diversity, we detected differences between dogs with CKD and the clinically healthy dogs across Jaccard (qualitative measures) and Bray-Curtis distances (quantitative measures) (*p* = 0.057, statistical trend and *p* = 0.035, respectively), whereas we did not detect differences between groups in the principal component analysis, based on unweighted and weighted UniFrac distance metrics (also qualitative and quantitative, considering bacterial phylogeny). Higher abundance of *Lachnospiraceae* family and *Blautia* bacterial species as well as *Ruminococcus gnavus* was observed in dogs with CKD when compared with healthy dogs. At the same time, a lower abundance of *Gammaproteobacteria* class, *Burkholderiales* order, *Sutterellaceae* family and *Sutterella* bacterial species was detected in dogs with CKD (Figure 3).

**Figure 4.**
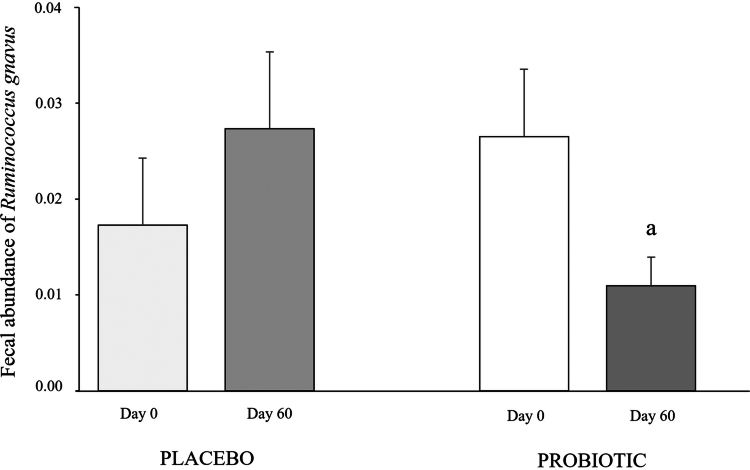
Changes in the abundance of *Ruminococcus gnavus* in dogs with chronic renal disease treated with probiotic or placebo at the beginning (day 0) and at the end (day 60) of the study. ^a^*p* = 0.041 vs day 0.

In a second step, we compared the effect of probiotic treatment on the abundance in bacterial populations that were up- or down-regulated in dogs with CKD. This analysis identified changes in the abundance of *Ruminococcus gnavus* but not in the other bacterial species. Adding the probiotic to the diet reduced the abundance of *Ruminococcus gnavus*, whereas consumption of the placebo did not change the abundance of this bacterial species (Figure 4).

## Discussion

### Microbiome

In human medicine, there is ample evidence of CKD-associated changes in intestinal microbiota that seem to be related to excretion of uremic toxins into the intestinal lumen (Vaziri et al. 2013; Ramezani and Raj 2014; Bartochowski et al. 2022). Renal dysfunction induces gut dysbiosis related to changes in intestinal pH, alterations in gut motility, and disrupted barrier integrity. As a consequence, there is a reduction of beneficial bacteria that produce short‑chain fatty acid–producing bacteria and an increase of harmful proteolytic bacteria that produce toxins (Alobaidi 2025). Thus, dysregulated gut microbiota is able to generate additional toxins, such as indoxyl sulfate, *p*‑cresyl sulfate and trimethylamine‑N‑oxide, which exert pro‑inflammatory, pro‑oxidative and pro‑fibrotic effects that may accelerate the progression of CKD and aggravate the clinical signs of the patient (Nii-Kono et al. 2007; Barreto et al. 2009; Gross et al. 2015; Nangaku et al. 2015; Tang et al. 2015; Stubbs et al. 2016). Increased intestinal permeability facilitates translocation of endotoxins that promote systemic inflammation and endothelial dysfunction contributing to renal injury. The combined effects of these mechanisms establish a vicious circle of gut dysbiosis and renal damage. This has led to the concept of gut–kidney axis as an important pathogenic factor in CKD and has heightened the interest in modulating intestinal microbiome in patients with CKD (Stavropoulou et al. 2021; Bartochowski et al. 2022).

Information about modifications of gut microbiome in pets with CKD is scant. Most research has been carried out in cats and the results of these studies showed moderate changes in gut microbiota that are reflected on several physiological parameters (Hall et al. 2020; Van Mulders et al. 2025a, 2025b). Van Mulders et al. (2025b) found an increase in gut-derived toxins (mainly indoxyl sulfate) in cats with CKD, while Hall et al. (2020) reported an increase in body mass and improved handling of indol compounds after consumption of an enriched diet for 8 weeks. In dogs, Ephraim and Jewell (2020) did not report marked differences in the fecal microbiome when comparing healthy dogs and dogs with early stages of CKD (IRIS I). However, a recent study that included dogs with more advanced CKD (IRIS I to IV) found dysregulated microbiota in dogs with kidney disease and the changes were more pronounced in dogs with severe CKD (Kim et al. 2023). These findings are similar to what we have found in dogs with CKD IRIS II and III. Thus, the available data support the contention that dysregulation of gut microbiome will occur in advanced stages of kidney disease. Similar to our findings, *Ruminococcus gnavus* has been reported to show higher abundance in humans with CKD (Lun et al. 2019).

*Ruminococcus gnavus* grows in dysbiotic and inflammatory environments and is associated with increased production of CKD‑related toxins. This species has been related to CKD progression through mechanisms involving mucin degradation and inflammation. Changes in mucin degradation contribute to disruption of the intestinal mucus layer, increasing the permeability of the epithelial barrier and thus facilitating microbial translocation and endotoxemia. Furthermore, *Ruminococcus gnavus* has been shown to modulate immune and metabolic pathways that amplify inflammatory signaling (Hong et al. 2024; Alobaidi 2025; Sokolovskaya et al. 2025).

A variety of probiotics have been proposed for use in human patients with CKD, including preparations containing the genera *Bifidobacterium*, *Lactobacilus* and *Enterococcus* (Koppe et al. 2015). In veterinary medicine, *Enterococcus lactis* is widely used as a probiotic in pigs, cattle and poultry (Krawczyk et al. 2021). Moreover, an *E. lactis* strain*, E. lactis* SF68, has been studied as a probiotic supplement to prevent and treat diarrhea in dogs and cats, with positive results (Bybee et al. 2011; Torres-Henderson et al. 2017).

In a study performed in cats with CKD, no significant changes in intestinal microbiota were found after treatment with *Enterococcus lactis* SF68 (Summers et al. 2019). In our study, feeding the probiotic resulted in attenuation of uremia-induced dysregulation of the gut microbiota. In particular, decreased abundance of *Ruminococcus gnavus* (a bacterial species that was up-regulated by CKD) was observed in the dogs that received the probiotic. *Ruminococcus gnavus* is one of the main bacteria that permits differentiation of intestinal microbiome between healthy humans and CKD patients (Lun et al. 2019; Garcia-Martinez et al. 2024). In addition, *Ruminococcus gnavus* has been shown to be implicated in the modulation of several metabolic pathways in humans with CKD (Liu et al. 2023). In agreement with our data, a synbiotic (pre- and probiotic) was shown to decrease *Ruminococcus gnavus* in the intestinal microbiota of human patients with CKD (Rossi et al. 2016).

### Biomarkers of renal disease

An improvement in renal function after treatment with probiotics has been documented in humans. Several studies have reported significant decreases in plasma concentrations of creatinine and urea and a delay in the decline of GFR in patients receiving probiotics (Ranganathan et al. 2010; Miranda Alatriste et al. 2014; Pavan 2014). In cats with CKD, a combination of prebiotics and probiotics has been reported to decrease azotemia in one paper (Palmquist 2006) and to not influence renal function in another paper (Rishniw and Wynn 2011). In an investigation in which dogs in stages II and III were supplemented with a probiotic formulation, serum creatinine concentrations did not change in the treated dogs, while it increased in the untreated controls. At the same time, treated dogs showed a significant increase in glomerular filtration rate (GFR) (Lippi et al. 2017). These data are in agreement with the decrease in SDMA and with the tendency to reduced proteinuria found in the dogs that received the probiotic in our study. The decrease in serum markers of GFR, like SDMA, after treatment with probiotics could be related to a reduction in the production of intestinal toxins and the subsequent attenuation of kidney damage. The increase in plasma cholesterol concentrations that was detected in both groups may reflect progression of renal disease (Li et al. 2022) and was not prevented by treatment with probiotic.

In veterinary medicine, uremic toxins have not received the same degree of attention as in human nephrology. The available data indicate that IxS is the most important gut-derived uremic toxin in cats and dogs. IxS concentrations have been shown to be consistently elevated in the plasma of uremic carnivores (Cheng et al. 2015; Liao et al. 2019; Summers et al. 2019; Hall et al. 2022; Van Mulders et al. 2025a, 2025b). In the dogs of the present study, IxS concentrations were elevated and in the range previously reported by other authors in dogs with CKD (Cheng et al. 2015; Liao et al. 2019). Several human studies have reported a reduction in uremic toxins after treatment with probiotics (Yu et al. 2022; Mitrea et al. 2023). In veterinary medicine, the effect of probiotics on uremic toxins has been investigated in cats supplemented with *Enterococcus lactis SF68* for 8 weeks, and no effect was found (Summers 2020). However, these data are difficult to interpret because in the same study, the probiotic formulation under investigation also failed to modify intestinal microbiota (Summers 2020). A more recent study in which a Lactobacillus mix was administered for 8 weeks demonstrated changes in the microbiome associated to a beneficial effect on systemic metabolite profile (Huang et al. 2025). In our study, the observed changes in gut microbiome were accompanied by a significant decrease in circulating IxS concentrations in dogs with CKD. IxS is known to induce oxidative stress that damages kidney cells and to promote tubulointerstitial fibrosis and glomerular sclerosis, thus contributing to accelerate the progression of kidney disease (Niwa 2010). Regarding cytokine analysis, values of IL2, IL10 and TNFα could not be measured because they were below the detection limit of the assay. This may reflect a technical limitation, and thus other procedures are needed to detect low values of these parameters. The small magnitude of the observed changes in biochemical parameters related to renal function in dogs receiving probiotic may question their biological significance. However, the differences were statistically significant even though the dogs were followed by a relatively short time. Further studies using a larger size sample in which the animals are monitored over a longer period are needed to clarify the clinical relevance of probiotic-induced changes in renal parameters.

Hypertension is a common complication in patients with CKD. Probiotics, when administered for a long period of time, may have a blood pressure-lowering effect in humans (Khalesi et al. 2014). To our knowledge, the effect of probiotics on blood pressure in uremic dogs has only been investigated in one study, in which only mean pressure was reported. Lippi et al. (2017) did not find changes in mean blood pressure after treatment with a probiotic formulation. Their data of mean arterial pressure are similar to ours. However, in our study, we found that probiotic administration prevented the increase in systolic blood pressure that was observed in the placebo group. Thus, it seems that supplementing the probiotic *Enterococcus lactis SF68* resulted in some beneficial effect, alleviating hypertension that could be secondary to a reduction in glomerulosclerosis as documented in other studies (Yang et al. 2021; Tungsanga et al. 2022; Zhang et al. 2022; Hou et al. 2024).

This study has some limitations. A larger sample size would have been desirable, but it is not easy to perform clinical studies in which owners show strict adherence to the research plan. Thus, backed by power analysis, we decided to limit the number of dogs to those for whom we could be sure the owners complied with all the requirements of the study. The study groups showed some heterogeneity in age, renal function and diet that is difficult to avoid when working with client-owned dogs, but none of these parameters showed statistically significant differences between groups. In any case, a larger sample size and a more homogeneous study population are likely to show more clearly the observed differences rather than to obscure them. Another limitation is the difficulty in obtaining meaningful results in some parameters (inflammatory profile and some uremic toxins) that were undetected by the techniques used for quantification.

In conclusion, the results of this study show that feeding a renal diet supplemented with *Enterococcus lactis SF68* probiotic formulation to dogs with CKD results in changes in intestinal microbiota that are associated to a decrease in plasma concentrations of SDMA and IxS and a reduction in systolic blood pressure.

## Supplementary Material

Supplementary Material Supp Table 1.docx

Supp_Table_2 (1).docx Supp_Table_2 (1).docx

## Data Availability

The data that support the findings of this study are available from the corresponding author upon reasonable request.
