# Supplementary material for: Supplementation with Enterococcus lactis (SF68) and its association with biochemical parameters and inflammatory biomarkers related to renal impairment in dogs with chronic kidney disease
Source: Vet Q. 2026 Apr 29;46(1):2665483. doi: 10.1080/01652176.2026.2665483 (PMC13130237; doi:10.1080/01652176.2026.2665483)
Supplement: Supplementary Material — Supp Table 1.docx [file TVEQ_A_2665483_SM2599.docx]

**Supp Table 1**. Ingredients and analytical constituents

| **Ingredients** | Meat and animal derivatives  Minerals |
| --- | --- |
| **Nutrition & Analytical Constituents (%)** | Protein: 55  Fat: 16,7  Crude ash: 11,6  Crude fibre: <0,5 |
| **Nutritional additives (mg/kg)** | Iron: 730  Iodine: 18  Copper: 110  Manganese: 340  Zinc: 1100  Selenium: 1 |
| **Vitamins (average level)** | Vitamin E: 6445 IU/Kg  Vitamin C: 1450 mg/Kg |
| **Live microencapsulated microorganisms** | *Enterococcus lactis* SF 68 NCIMB 10415: 5x10^11^ CFU/kg |
