# Supplementary material for: Supplementation with Enterococcus lactis (SF68) and its association with biochemical parameters and inflammatory biomarkers related to renal impairment in dogs with chronic kidney disease
Source: Vet Q. 2026 Apr 29;46(1):2665483. doi: 10.1080/01652176.2026.2665483 (PMC13130237; doi:10.1080/01652176.2026.2665483)
Supplement: Supp_Table_2 (1).docx [file TVEQ_A_2665483_SM2598.docx]

| **Nutrients (%)** | **Royal Canin Renal** | **Purina**  **NF Renal** | **Hill’s**  **Kidney care (k/d)** |
| --- | --- | --- | --- |
| Crude protein | 14.00 | 14.10 | 13.9 |
| Crude oils and fats | 18 | 15.70 | 20.5 |
| Carbohydrates | 52.6 | 63.20 | 51.2 |
| Crude fiber | 2.4 | 2.20 | 1.5 |
| Crude ash | 4.0 | 4.90 | 4.4 |
| Calcium | 0.4 | 0.75 | 0.63 |
| Phosphorus | 0.2 | 0.43 | 0.27 |
| Sodium | 0.35 | 0.2 | 0.16 |
| Potassium | 0.60 | 0.8 | 0.68 |
| Magnesium | 0.09 | 0.09 | 0.1 |
| Omega 6 | 4.18 | 2.5 | 3.65 |
| Omega 3 | 1.11 | 0.4 | 1.11 |
| EPA+DHA | 0.55 | 0.22 | 0.54 |
| Vitamin A (UI/kg) | 15500 | 31000 | 10965 |
| Vitamin D (UI/Kg) | 1000 | 1000 | 877 |
| Vitamin E (UI/Kg) | 500 | 350 | 584 |
| Metabolizable energy (Kcal/100 g) | 385 | 390 | 402 |

**Supp Table 2**. Analytical constituents of the three diets used in the study.
